# Botulinum toxin for chronic migraine

**DOI:** 10.1007/s00702-025-03055-2

**Published:** 2025-11-06

**Authors:** Lejla Paracka, Dirk Dressler

**Affiliations:** 1 Movement Disorders Section, Department of Neurology, Hannover Medical School, Carl-Neuberg-Str. 1, 30625 Hannover, Germany; 2 Neurotoxin Research Center, Tongji University Medical School, Shanghai, China

**Keywords:** Botulinum toxin, Chronic migraine, Review, Safety, Efficacy

## Abstract

Chronic migraine (CM) is a common primary headache occurring on 15 or more days per month and for more than three months. It presents a considerable therapeutic challenge. Botulinum toxin (BoNT) has emerged as a promising treatment for CM, particularly in patients unresponsive to standard pharmacological therapy or experiencing adverse effects. Its therapeutic benefit is based on blocking the release of nociceptive neuropeptides modulating pain pathways. Clinical efficacy and safety of BoNT have been demonstrated in multiple randomized controlled CM trials showing a significant reduction of headache frequency. This review provides an overview of efficacy and safety of BoNT for CM.

## Introduction

Migraine is a common neurological disorder characterised by recurrent, usually unilateral headache attacks usually lasting between 4 and 72 h. They are often typically pulsating and throbbing in nature, are moderate to severe in intensity and worsened by physical activity. They are often accompanied by nausea, vomiting and extreme sensitivity to light and sound. Transient neurological deficits called aura may precede or accompany the headache attacks. They may include visual disturbances, such as flashes of light or blind spots, sensory disturbances and difficulties speaking.

Chronic migraine (CM) is defined as headaches occurring on 15 or more days per month for more than three months with at least 8 of those days presenting with migraine-like features, with or without aura (International Classification of Headache Disorders, 3rd edition) (ICHD-3 2018).

## Epidemiology

In 2021 global prevalence of migraine was 1,2 billion cases (95% UI, 1.0-1.3), with age standardised prevalence rate of approximately 15% (GBD 2021 Headache collaborators et al. 2025). It accounts for 4.9% of global ill health, measured in years lived with disability. Migraine is the second leading cause of disability worldwide and the leading cause among young adults (Steiner et al. 2023). CM ranks as the third-highest cause of disability globally in both males and females under the age of 50 (Steiner et al. 2023). Although prevalence estimates for CM vary, the most reliable estimate places its global prevalence at about 1% (Natoli et al. 2010).

### Treatment of chronic migraine

Treatment of CM is challenging and comprises of acute and preventive treatments. For acute CM attacks, non-steroidal anti-inflammatory drugs and triptanes are usually used. Preventive or prophylactic treatment does not prevent migraine as such, but reduces the intensity, frequency and duration of migraine attacks. Conventionally, antihypertensive, antidepressive and antiepileptic drugs have been used. However, they are often ineffective and adverse effects may restrict their use. The introduction of onabotulinumtoxinA (ONA, Botox^®^, AbbVie-Allergan) has improved preventive migraine therapy considerably. Recently, new generations of oral small molecule calcitonin gene-related peptide (CGRP) antagonists (gepants) (Li et al. 2023; Barbanti et al. 2025), lasmiditan, a potent and selective agonist of the 5-HT_1F_ receptor and monoclonal antibodies targeting CGRP or its receptor have been introduced (Pellesi et al. 2024; Puledda et al. 2020).

Non-pharmacological measures such as stress and weight reduction and sufficient sleep may prevent migraine attacks and may reduce the risk of transformation of episodic migraine to CM (Escher et al. 2017; Schwedt 2014).

German guidelines for migraine treatment recommend ONA treatment in patients non-responsive to at least two previous pharmacological therapies. The patients should undergo two to three treatment cycles, before efficacy is decided (Diener et al. 2022).

## Botulinum toxin

Botulinum toxin (BoNT) is a potent neurotoxin produced by Clostridium botulinum. Its clinical use started, when Dr Alan B Scott introduced it for the treatment of strabismus. Over the years, BoNT has become a highly effective and safe therapy for a large number of conditions (Dressler 2012) including dystonia (Dressler et al. 2021a; Simpson et al. 2016), spasticity in adults (Dressler et al. 2021a, b) and infants (Multani et al. 2019), hypersalivation (Paracka et al. 2019; Jost et al. 2019), hyperhidrosis (Heckmann et al. 2001) and hemifacial spasm (Park et al. 2023).

In nerve terminals, vesicles containing neurotransmitters are transported to the synaptic cleft by the SNARE (soluble N-ethylmaleimide-sensitive fusion attachment protein receptor) protein complex, containing SNAP-25 (Alonge et al. 2024). This protein is cleaved and inactivated by BoNT. In motor nerves, BoNT blocks the neuromuscular junction and produces paresis. Furthermore, in animal model studies it has been demonstrated the axonal migration and transcytosis of BoNT to the trigeminal ganglion and central nervous system, cleaving SNAP-25, highlighting its potential functional implications (Antonucci et al. 2008).

In nociceptive pathways, conveying pain information from the periphery to the central nervous system, and in inflammation mechanisms, BoNT is thought to block other SNARE dependent neurotransmitters including CGRP, substance P and glutamate. It may also modulate pain-associated membrane-channels by transient receptor potential cation channel subfamily V member 1 (TRPV1). Nerve endings of sensory neurons located in the trigeminal and cervical ganglia are overactive in migraine. Potential inhibition of these sensory nerve endings by BoNT, may reduce the number of pain signals reaching the brain (Burstein et al. 2020). Some preclinical studies have shown the role of BoNT in reducing periorbital hypersensitivity in inflammation models, by reducing CGRP, while others report contradictory findings (Reducha et al. 2024; Lackovic et al. 2015).

## Botulinum toxin for treatment of chronic migraine

In 2010, ONA has shown its efficacy and safety for CM therapy in the PREEMPT 1 and PREEMPT 2 (Research Evaluating Migraine Prophylaxis Therapy) trials (Aurora et al. 2010; Diener et al. 2010). Subsequently, it was approved by the US Food and Drug Administration and the European Medicines Agency for the prophylaxis of CM. Its use was also endorsed by the National Institute for Health and Care Excellence (NICE) in 2012.

The PREEMPT 1 and PREEMPT 2 trials were 24-week, double-blind, parallel group, placebo-controlled studies followed by 32-week, open-label studies. A total of 1384 patients were included in these studies (PREEMP 1: *n* = 679, PREEMPT 2: *n* = 705). The subjects were treated with a fixed scheme of ONA 155MU to 195MU spread over 31 injection sites in 7 target muscles of the head (Table 1). In PREEMPT 1 (Diener et al. 2010), although there was no group difference for the primary endpoint, significant reductions from baseline were observed for secondary endpoint in reduction of headache (*p* = 0.006) and migraine days (*p* = 0.002), cumulative hours of headache on headache days and frequency of moderate/severe headache days. In PREEMPT 2 (Aurora et al. 2010), ONA was statistically significantly superior to placebo for the primary endpoint, frequency of headache days per 28 days relative to baseline (*p* < 0.001), as well as in all secondary endpoint comparisons. Adverse effects including muscle weakness, ptosis and neck pain were rare and fully reversible.

After the PREEMPT studies, several real-life studies followed. One of the largest study was the COMPEL (Chronic Migraine OnabotulinuMtoxinA Prolonged Efficacy Open Label) study (Blumenfeld et al. 2018), an international, multicentre, open-label study. 716 adults with CM received 155MU of ONA in a fixed injection scheme according to PREEMPT (Table 1). The injection scheme consisted of 31 injection sites in seven head and neck muscles every 12 weeks (± 7 days) for 9 treatment cycles or 108 weeks. The primary outcome parameter was headache day reduction at 108 weeks. Secondary outcome parameters were headache day reductions at 60 weeks and change in the 6-item Headache Impact Test (HIT-6) score. By 60 and 108 weeks, a significant reduction in headache days (*p* < 0.0001) and improvements in HIT-6 (*p* < 0.0001) scores were demonstrated. The most common adverse effect was neck pain.

Another large CM study was the REPOSE (real-life use of botulinum toxin for the symptomatic treatment of adults with chronic migraine, measuring healthcare resource utilisation, and patient-reported outcomes observed in practice) study, a prospective long-term, multicentre European study. In this study, 641 CM patients were enrolled and observed for 24 months. Outcome measures included ONA injection practices (Table 1), headache-day frequency, Migraine-Specific Quality-of-Life Questionnaire (MSQ), EuroQol 5-Dimension Questionnaire (EQ-5D). Headache-day frequency was reduced at injection visit 8 (*p* < 0.001). Each MSQ domain (restrictive, preventive and emotional) was significantly reduced from baseline throughout each injection visit (*p* < 0.001). The median EQ-5D total and health state scores were significantly improved from baseline throughout each injection visit. Eyelid ptosis, neck pain and musculoskeletal stiffness were the most reported adverse events (Ahmed et al. 2019).

In another prospective analysis of 254 patients (Khalil et al. 2014), ONA significantly reduced the number of headache and migraine days and significantly increased the number of headache free days. ONA also improved patient’s quality of life.

In a retrospective real-life data study of 115 patients with CM and acute medication overuse headache who were treated with ONA, 62% of patients discontinued medication overuse within one year. Among those receiving preventive therapy (topiramate and beta-blocker), in 49% these were discontinued. In 19%, ONA administration was delayed to the fourth or fifth month and in 10%, it was temporarily stopped. Finally, in 16% ONA was discontinued due to lack of efficacy beyond first year of treatment. These results show, that a higher consumption of analgetics at baseline was negatively associated with good response after one year of treatment (Aicua-Rapun et al. 2016; Baraldi et al. 2023).

An Italian study (Negro et al. 2015) compared the efficacy and safety of ONA 195MU versus 155MU for the treatment of CM and medication overuse headache during a 2-year period. This study demonstrated the superior efficacy of ONA 195MU compared to 155MU with a similar safety and tolerability profile.

Later, another retrospective study (Zandieh & Cutrer 2023) found, that ONA reduces headache days and severe headache days.

Another study (Guerzoni et al. 2015) showed that ONA is an effective treatment to reduce the headache-related disability and improve patients’ quality of life, depressive and anxiety symptoms, when patients are treated regularly and consistently every three months. Therapy discontinuation leads to a general worsening of health-related quality of life.

Another multicentre prospective study (Corbelli et al. 2023) demonstrated a sustained efficacy in patients initially characterised as ‘partially responders’ in the first year, when the treatment continued over the second year. ONA treatment was beneficial for long lasting CM prevention with a significant effect over 4 years of observation (Santoro et al. 2020).

In the multicentre, randomised, open-label prospective FORWARD study (Rothrock et al. 2019), 282 adults with CM were randomized (1:1) to ONA 155MU every 12 weeks for three cycles or topiramate immediate release 50-100 mg/d to week 36. Primary outcome measure was proportion of patients achieving greater than 50% reduction in headache days. A significantly higher proportion of patients randomised to ONA experienced greater than 50% reduction in headache frequency compared to those randomised to topiramate (40% versus 12% respectively; adjusted OR 4.8 (95% CI, 2.7–9.1).

Similar results were shown in randomised controlled studies with Valproate 250 mg (Blumenfeld et al. 2008) and Amitriptyline 25-50 mg (Magalhaes et al. 2010).

In a recent retrospective multicentre study of 316 patients (Wang et al. 2024), CM patients treated with calcitonin gene-related peptide monoclonal antibody (CGRP Ab) were compared to those treated with ONA. At 6 months, CGRP Ab treatment was associated with greater decrease in monthly migraine day (MMDs) (-13 versus − 8.7 days/month, *p* < 0.001) and a higher greater than 50% responder rate (74.7% versus 50.7%, *p* < 0.001) compared with ONA. Both injectables were effective and well tolerated. However, this study had some limitations. This was a retrospective study and certain patient reported outcomes such as Migraine Disability Assessments and subjective ratings on overall effectiveness were not available for all patients. On the other hand, since the ONA treatment was compared to baseline, one could argue that the optimal effect hasn’t been reached yet after the second treatment.

## Adverse effects

Adverse events during the treatment with ONA in CM are mostly local. Most often, adverse events are muscle pain, injection site pain and swelling due to the mechanical stress during the procedure. Muscular weakness of trapezius muscle and rarely ptosis can occur. However, these are transitory and rarely lead to treatment termination. Post injection headaches, flu-like symptoms and malaise are rare, but have been described and might be related to systemic effects of BoNT (Baraldi et al. 2023).

Various studies have shown that ONA treatment of CM is safe and well tolerable even after long-term application (Guerzoni et al. 2017 ). So far, no interactions of BoNT with drugs have been reported.

## Limitations

A key limitation of botulinum toxin study is the high placebo response, a phenomenon that is observed in other migraine treatment trials. Additionally, BoNT studies is difficult to blind as participants can recognise group allocations based on visible aesthetic changes. Adequate blinding represent a major methodological challenge in botulinum toxin studies. Active placebo injections, delayed start or crossover design may help distinguish true therapeutic effect from placebo responses.

## Conclusions

ONA is an effective treatment of CM. It can reduce headache days, pain intensity and can improve quality of life. Its adverse effect profile is advantageous, as it does not participate in systemic metabolism. Future studies should optimise dosing and target muscle selection. Exploring BoNT’s analgesic effects in other pain conditions seems warranted.

**Table 1:** PREEMPT scheme for botulinum toxin therapy of chronic migraine

Target muscle	ONA dose	Additional ONA dose (optional)
M. procerus	5MU (1 site)	
M. corrugator (bil.)	10MU (2 sites)	
M. frontalis (bil.)	20MU (4 sites)	
M. occipitalis (bil.)	30MU (6 sites)	10MU (2 sites)
M. temporalis (bil.)	40MU (8 sites)	10MU (2 sites)
M. trapezius (bil.)	30MU (6 sites)	20MU (4 sites)
M. paraspinalis (bil.)	20MU (4 sites)	
Total	155MU	40MU

*ONA* onabotulinumtoxinA, *MU* mouse units, *M.* musculus

## Data Availability

No datasets were generated or analysed during the current study.

## References

[CR1] Ahmed F, Gaul C, García-Moncó JC, Sommer K, Martelletti P, REPOSE principal investigators (2019) an open-label prospective study of the real-life use of OnabotulinumtoxinA for the treatment of chronic migraine: the REPOSE study. J Headache Pain ;20:26. 10.1186/s10194-019-0976-1 30845917 10.1186/s10194-019-0976-1 PMC6734221

[CR2] Aicua-Rapun I, Martínez-Velasco E, Rojo A, Hernando A, Ruiz M, Carreres A, Porqueres E, Herrero S, Iglesias F, Guerrero AL (2016) Real-life data in 115 chronic migraine patients treated with onabotulinumtoxin A during more than one year. J Headache Pain 17:112. 10.1186/s10194-016-0702-1 27957623 10.1186/s10194-016-0702-1 PMC5153399

[CR3] Alonge P, Brighina F, Maccora S, Pilati LD, Marco S, Ventimiglia D, Maggio B, Cutrò I, Camarda C, Torrente A (2024) Beyond pain: the effects of OnabotulinumtoxinA therapy on sensitization and interictal symptoms in chronic migraine. Toxins 16:203. 10.3390/toxins16050203 38787055 10.3390/toxins16050203 PMC11125997

[CR4] Antonucci F, Rossi C, Gianfranceschi L, Rossetto O, Caleo M (2008) Long-distance retrograde effects of botulinum neurotoxin A. J Neurosci. 2;28:3689-96. 10.1523/JNEUROSCI.0375-08.2008 10.1523/JNEUROSCI.0375-08.2008 PMC6671090 18385327

[CR5] Aurora SK, Dodick DW, Turkel CC, DeGryse RE, Silberstein SD, Lipton RB, Diener HC, Brin MF, PREEMPT 1 chronic migraine study group (2010) OnabotulinumtoxinA for treatment of chronic migraine: results from the double-blind, randomized, placebo-controlled phase of the PREEMPT 1 trial. Cephalalgia 30:793–803

[CR6] Baraldi C, Lo Castro F, Ornello R, Sacco S, Pani L, Guerzoni S (2023) OnabotulinumtoxinA: still the present for chronic migraine. Toxins 15:59. 10.3390/toxins15010059 36668879 10.3390/toxins15010059 PMC9865956

[CR7] Barbanti P, Egeo G, Pistoia F, Aurilia C, Scatena P, Rinalduzzi S, Strumia S, Salerno A, Frediani F, Galli A, Autunno M, Di Clemente L, Zucco M, Albanese M, Bono F, Bruno P, Borrello L, Messina S, Doretti A, Ranieri A, Camarda C, Vecchio R, Drago V, Fiorentini G, Tomino C, Bonassi S, Torelli P, mannocci A; Italian migraine registry (I-GRAINE) study group (2025) GIANT: a prospective, multicenter, real-world study on the effectiveness, safety, and tolerability of Atogepant in migraine patients with multiple therapeutic failures. J Headache Pain 19;26:122. 10.1186/s10194-025-02068-2 10.1186/s10194-025-02068-2 PMC12090485 40389834

[CR8] Blumenfeld AM, Schim JD, Chippendale TJ (2008) Botulinum toxin type A and divalproex sodium for prophylactic treatment of episodic or chronic migraine. Headache 48:210–220 18047502 10.1111/j.1526-4610.2007.00949.x

[CR9] Blumenfeld AM, Stark RJ, Freeman MC, Orejudos A, Manack Adams A (2018) Long-term study of the efficacy and safety of OnabotulinumtoxinA for the prevention of chronic migraine: COMPEL study. J Headache Pain 19:13. 10.1186/s10194-018-0840-8 29404713 10.1186/s10194-018-0840-8 PMC5799088

[CR10] Burstein R, Blumenfeld AM, Silberstein SD, Manack Adams A, Brin MF (2020) Mechanism of action of OnabotulinumtoxinA in chronic migraine: A narrative review. Headache 60:1259–1272 32602955 10.1111/head.13849 PMC7496564

[CR12] Diener HC, Förderreuther S, Kropp P (2022) Therapie der Migräneattacke und prophylaxe der Migräne, S1-Leitlinie. In: Deutsche Gesellschaft für Neurologie (Ed), Leitlinien für Diagnostik und Therapie in der Neurologie

[CR11] Diener HC, Dodick DW, Aurora SK et al (2010) OnabotulinumtoxinA for treatment of chronic migraine: results from the double-blind, randomized, placebo-controlled phase of the PREEMPT 2 trial. Cephalalgia 30:804–814 20647171 10.1177/0333102410364677

[CR13] Dressler D (2012) Clinical applications of botulinum toxin. Curr Opin Microbiol 15:325–336 22770659 10.1016/j.mib.2012.05.012

[CR14] Dressler D, Adib Saberi F, Rosales RL (2021a) Botulinum toxin therapy of dystonia. J Neural Transm 128:531–537 33125571 10.1007/s00702-020-02266-z PMC8099791

[CR15] Dressler D, Altavista MC, Altenmueller E, Bhidayasiri R, Bohlega S, Chana P, Chung TM, Colosimo C, Fheodoroff K, Garcia-Ruiz PJ, Jeon B, Jin L, Kanovsky P, Milanov I, Micheli F, Orlova O, Pandey S, Pirtosek Z, Relja M, Rosales R, Sagástegui-Rodríguez JA, Shahidi GA, Timerbaeva S, Wan X, Walter U, Saberi FA (2021b) Consensus guidelines for botulinum toxin therapy: general algorithms and dosing tables for dystonia and spasticity. J Neural Transm 128:321–335 33635442 10.1007/s00702-021-02312-4 PMC7969540

[CR16] Escher CM, Paracka L, Dressler D, Kollewe K (2017) Botulinum toxin in the management of chronic migraine: clinical evidence and experience. Ther Adv Neurol Disord 10:127–135 28382110 10.1177/1756285616677005 PMC5367647

[CR17] GBD 2021 Headache Collaborators (2025) Global, regional, and National burden of headache disorders, 1990–2021, with forecasts to 2050: A global burden of disease study 2021. Cell Rep Med 18:102348. doi: 10.1016/j.xcrm.2025.102348 10.1016/j.xcrm.2025.102348 PMC12629824 40972580

[CR19] Guerzoni S, Pellesi L, Baraldi C, Pini LA (2015) Increased efficacy of regularly repeated cycles with OnabotulinumtoxinA in MOH patients beyond the first year of treatment. J Headache Pain 17:48. 10.1186/s10194-016-0634-9 27146068 10.1186/s10194-016-0634-9 PMC4856636

[CR18] Guerzoni S, Pellesi L, Baraldi C, Cainazzo MM, Negro A, Martelletti P, Pini LA (2017) Long-term treatment benefits and prolonged efficacy of OnabotulinumtoxinA in patients affected by chronic migraine and medication overuse headache over 3 years of therapy. Front Neurol 8:586. 10.3389/fneur.2017.00586 29163347 10.3389/fneur.2017.00586 PMC5676047

[CR21] Headache Classification Commitee of the International Headache Society (2018) International Classification of Headache Disorders, 3rd edition. Cephalalgia 38:1-211 10.1177/0333102417738202 29368949

[CR20] Heckmann M, Ceballos-Baumann AO, Plewig G (2001) The hyperhidrosis study Group. Botulinum toxin A for axillary hyperhidrosis (excessive sweating). N Engl J Med 344:488–493 11172190 10.1056/NEJM200102153440704

[CR22] Jost WH, Friedman A, Michel O, Oehlwein C, Slawek J, Bogucki A, Ochudlo S, Banach M, Pagan F *Flatau-Baqué B, Csikós J, Cairney CJ, blitzer A (2019)* SIAXI. Placebo-controlled, randomized, double-blind study of incobotulinumtoxina for sialorrhea. Neurology 92:e1982–e1991. 10.1212/WNL.0000000000007368 10.1212/WNL.0000000000007368 PMC6511076 30918101

[CR23] Khalil M, Zafar HW, Quarshie V, Ahmed F (2014) Prospective analysis of the use of OnabotulinumtoxinA (BOTOX) in the treatment of chronic migraine; real-life data in 254 patients from Hull, UK. J Headache Pain 15:54. 10.1186/1129-2377-15-54 25178393 10.1186/1129-2377-15-54 PMC4166400

[CR24] Lacković Z, Filipović B, Matak I, Helyes Z (2016) Activity of botulinum toxin type A in cranial dura: implications for treatment of migraine and other headaches. Br J Pharmacol 173:279–291. 10.1111/bph.13366 26493010 10.1111/bph.13366 PMC5341233

[CR25] Li D, Abreu J, Tepper SJ (2023) A brief review of gepants. Curr Pain Headache Rep 27:479–488 37531032 10.1007/s11916-023-01142-1

[CR26] Magalhaes E, Menezes C, Cardeal M, Melo A (2010) Botulinum toxin type A versus amitriptyline for the treatment of chronic daily migraine. Clin Neurol Neurosurg. 112:463–466 20399553 10.1016/j.clineuro.2010.02.004

[CR27] Multani I, Manji J, Hastings-Ison T, Khot A, Graham K (2019) Botulinum toxin in the management of children with cerebral palsy. Pediatr Drugs 21:261–281 10.1007/s40272-019-00344-8 PMC6682585 31257556

[CR28] Natoli JL, Manack A, Dean B, ButlerQ, Turkel CC, Stovner L, Lipton RB (2010) Global prevalence of chronic migraine: a systematic review. Cephalalgia 30:599–609 19614702 10.1111/j.1468-2982.2009.01941.x

[CR29] Negro A, Curto M, Lionetto L, Martelletti P ((2015)) A two years open-label prospective study of OnabotulinumtoxinA 195 U in medication overuse headache: a real-world experience. J Headache Pain 17:1. 10.1186/s10194-016-0591-3 26792662 10.1186/s10194-016-0591-3 PMC4720620

[CR30] NICE technology appraisal guidance 260 (2012) Botulinum toxin type A for the prevention of headaches in adults with chronic migraine. Available from ) Botulinum toxin type A for the prevention of headaches in adults with chronic migraine. Available from https://www.nice.org.uk/guidance/ta260

[CR31] Paracka L, Kollewe K, Klietz M, Petri S, Dressler D (2019) IncobotulinumtoxinA for hypersalivation in patients with amyotrophic lateral sclerosis: an open-label single-centre study. J Neural Transm 126:1341–1345 31317261 10.1007/s00702-019-02044-6

[CR32] Park CK, Lim SH, Park K ((2023)) Clinical application of botulinum toxin for hemifacial spasm. Life 13:1760. 10.3390/life13081760 37629617 10.3390/life13081760 PMC10455826

[CR33] Pellesi L, Garcia-Azorin D, Rubio-Beltrán E, Ha WS, Messina R, Ornello R, Petrusic I, Raffaelli B, Labastida-Ramirez A, Ruscheweyh R, Tana C, Vuralli D, Waliszewska-Prosół M, Wang W, Wells-Gatnik W (2024) combining treatments for migraine prophylaxis: the state-of-the-art. J Headache Pain 25:214. 10.1186/s10194-024-01925-w 10.1186/s10194-024-01925-w PMC11619619 39639191

[CR34] Puledda F, Younis S, Huessler E-M, Haghdoost F, Lisicki M, Goadsby PJ, Tassorelli C (2023) Efficacy, safety and indirect comparisons of lasmiditan, rimegepant, and ubrogepant for the acute treatment of migraine: A systematic review and network meta-analysis of the literature. Cephalalgia 43:3331024231151419. 10.1177/03331024231151419 36786357 10.1177/03331024231151419

[CR35] Reducha PV, Bömers JP, Edvinsson L, Haanes KA (2024) The impact of the migraine treatment OnabotulinumtoxinA on inflammatory and pain responses: insights from an animal model. Headache 64:652–662. 10.1111/head.14726. Epub 2024 May 3. 38700141 10.1111/head.14726

[CR36] Rothrock JF, Adams AM, Lipton RB, Silberstein SD, Jo E, Zhao X, Blumenfeld AM (2019) FORWARD study investigative group. FORWARD study: evaluating the comparative effectiveness of OnabotulinumtoxinA and topiramate for headache prevention in adults with chronic migraine. Headache 59:1700–1713 31559634 10.1111/head.13653 PMC6899480

[CR37] Santoro A, Copetti M, Miscio AM, Leone MA, Fontana A (2020) Chronic migraine long-term regular treatment with onabotulinumtoxina: a retrospective real-life observational study up to 4 years of therapy. Neurol Sci 41:1809–1820 32052306 10.1007/s10072-020-04283-y PMC7359167

[CR38] Schwedt T (2014) Chronic migraine. Brit Med J 348:g1416. 10.1136/bmj.g1416 24662044 10.1136/bmj.g1416

[CR39] Simpson DM, Hallett M, Ashman EJ, Comella CL, Green MW, Gronseth GS, Yablon SA (2016) Practice guideline update summary: botulinum neurotoxin for the treatment of blepharospasm, cervical dystonia, adult spasticity, and headache: report of the guideline development subcommittee of the American academy of neurology. Neurology 86:1818–1826 27164716 10.1212/WNL.0000000000002560 PMC4862245

[CR40] Steiner TJ, Stovner LJ (2023) Global epidemiology of migraine and its implications for public health and health policy. Nat Rev Neurol 19:109–117 36693999 10.1038/s41582-022-00763-1

[CR41] Wang YF, Yang FC, Chen LA, Chang TY, Su HC, Yang CP, Tu YH, Tzeng YS, Chen SP, Fuh JL, Lai KL, Ling YH, Chen WT, Wang SJ (2024) Comparative effectiveness and tolerability of calcitonin gene-related peptide (CGRP) monoclonal antibodies and OnabotulinumtoxinA in chronic migraine: A multicenter, real-world study in Taiwan. Eur J Neurol 31:e16372. 10.1111/ene.1637 38837528 10.1111/ene.16372 PMC11295178

[CR42] Zandieh A, Cutrer FM (2022) OnabotulinumtoxinA in chronic migraine: is the response dose dependent? BMC Neurol 22:218. 10.1186/s12883-022-02742-x 35698027 10.1186/s12883-022-02742-x PMC9190093

